# The Analysis of Etiology and Risk Factors for 192 Cases of Neonatal Sepsis

**DOI:** 10.1155/2017/8617076

**Published:** 2017-07-03

**Authors:** Ting Xiao, Li-Ping Chen, Hui Liu, SiSi Xie, Yan Luo, Ding-Chang Wu

**Affiliations:** ^1^Department of Clinical Laboratory, Fujian Longyan First Hospital, Longyan First Affiliated Hospital, Fujian Medical University, Longyan, Fujian 364000, China; ^2^Department of Neonatal Unit, Fujian Longyan First Hospital, Longyan First Affiliated Hospital, Fujian Medical University, Longyan, Fujian 364000, China

## Abstract

This study aimed to investigate the etiology and risk factors of neonatal sepsis. A retrospective analysis was conducted on 192 patients with sepsis from August 2013 to March 2015. One hundred and six healthy neonates were used as the control group. Logistic regression was used to analyze the risk factors and ROC curve analysis performed in laboratory which indicated a significant correlation. The results of univariate analysis showed that postnatal age, body weight, and parity were significantly related to neonatal sepsis (*P* < 0.5). Logistic regression analysis demonstrated that postnatal age and parity are independent risk factors for neonatal sepsis (OR were 1.176 and 0.692, resp., *P* < 0.001). The maximum area underneath the curve (ROC^AUC^) of soluble CD14 (sCD14-ST), which was the most indicative biomarker of sepsis diagnostically, was 0.953 with sensitivity and specificity of 93.8% and 84.9%, respectively.* Escherichia coli, Staphylococcus aureus,* and* Streptococcus agalactiae* were the main bacterial strains causing neonatal sepsis, while postnatal age was an independent risk factor for the onset of disease. sCD14-ST could be a potential useful diagnostic marker for pediatric sepsis.

## 1. Introduction

Sepsis, a common critical illness in the neonatal intensive care unit, is a systemic inflammatory response syndrome (SIRS) in infectious diseases. Its clinical manifestations are nonspecific. Sepsis is easily misdiagnosed, because newborns are very young and their organs are still developing [1–3]. Sepsis is often a consequence of a respiratory tract infection or burns. According to WHO, severe infections have caused 60% of deaths in children under the age of five, in which 6 million newborns and children die of sepsis annually [4].

A variety of biological indicators, such as serum C-reactive protein (CRP), and numeration of leukocyte (WBC) have been used in the laboratory to diagnose neonatal sepsis. Pediatric critical illness scoring system is often used to assess the severity and prognosis of sepsis, but there are still limitations [5]. For example, CRP can appear in almost all inflammation-related reactions, and WBC can display the characteristic of hysteresis. WHO reported that the morbidity of patients diagnosed with severe sepsis in the neonatal intensive care unit is higher than 15%. In 2009, the annual report for inpatient newborns in countries like Australia showed that the incidence of neonatal sepsis in grade III neonatal intensive care unit was 7.7%, while it was 49.1% and 73.6% in infants younger than 28 and 32 weeks, respectively [6]. It can be seen that age is one of the important factors influencing neonatal sepsis and that its incidence will decline with an increase in age [7]. Meanwhile, Martin et al. also found that age was an independent risk factor for the prognosis of sepsis in adults [8].

Nevertheless, there are few reports about the predictive factors of neonatal sepsis in China. In this study, we made the assumption that neonatal sepsis is correlated with age. We reviewed the clinical data of 192 cases of neonatal sepsis, summarized the risk factors, and provided clinical guidelines for monitoring its onset.

## 2. Materials and Methods

### 2.1. Patient Information

A retrospective analysis was performed on 192 neonatal sepsis cases from the neonatal intensive care unit (NICU) in Longyan First Hospital, Fujian province, diagnosed in the period from August 2013 to March 2015. All sepsis cases were consistent with the diagnostic criteria developed by international pediatric specialists in 2005 and based on the physiological characteristics of children of different ages (including newborns) [9]. Patients with congenital immune diseases or congenital malformations or those who received immunosuppressive therapy and were treated in other hospitals for more than 3 days were excluded from the study.

At least two of the following four SIRS diagnostic criteria for children were identified: (i) central body temperature greater than 38.5°C or less than 36°C; (ii) tachycardia, the average heart rate greater than two standard deviations of the normal value in the same age group (no external stimuli, chronic drugs, or pain stimulation), or bradycardia occurring in patients less than 1 year old, or the average heart rate smaller than the tenth percentile of the value in the same age group (no external vagal stimulation and congenital heart disease and no use of *β*-blockers), or nonexplanatory sustained slowdown observed over 0.5 h; (iii) the mean respiratory frequency was greater than two standard deviations of the normal value in all age groups, or mechanical ventilation was needed due to the acute course of the disease (no neuromuscular disease and no correlation with general anesthesia); (iv) increased or decreased white blood cell count (neutropenia that was not secondary to chemotherapy) or immature neutrophil value greater than 0.10.

Infection refers to the existence of suspected or confirmed infections caused by any pathogen (positive culture, tissue staining, or PCR) or a clinical syndrome that is highly related to infection. Evidence of infection includes a clinical examination, X-ray radiography, or positive laboratory results (such as white blood cells in sterile liquid, visceral perforation, persistent pneumonia confirmed by chest X-ray, petechiae or purpura rash, and purpura fulminans).

106 healthy newborns were used as the control group. The inclusion criteria were as follows: the patients were free of symptoms of clinical acute or chronic infections and congenital diseases and did not receive any medications.

This study was approved by and conformed to the relevant provisions of the Ethics Committee of Longyan First Affiliated Hospital of Fujian Medical University.

### 2.2. Specimen Collection

2 ml EDTA anticoagulant venous blood and 5 ml separated gel procoagulant venous blood were collected from all subjects. 2~3 ml venous blood from each side was extracted under sterile conditions upon patient admission. Following this, the blood was injected into the enrichment broth in a blood culture flask sterilized with 75% alcohol. BACTEC FX from Becton, Dickinson and Company (BD, NJ, USA) was used to perform the culture. If rosy precipitate was observed, or if the medium became cloudy within 72 h, the blood was transferred to a culture plate. Specimen transferring BD Phoenix 100 was used to perform typing identification, as well as the drug sensitivity test for bacteria, and combined with clinical symptoms to determine true and false positive outcomes.

2 ml EDTA anticoagulant venous blood and 5 ml separated gel procoagulant venous blood were obtained from all healthy newborns to be used as the control.

### 2.3. Collection of Clinical Data

Clinical data were collected from the newborns including the name, gender, age, gestational age, parity, and birth weight. All cases were grouped according to the onset of sepsis.

The determination of sCD14-ST, CRP, and WBC was performed 10 times using the PATHFAST cardiac marker immune analyzer supplied with a kit from Mitsubishi (chemiluminescence immunoassay), a Backman 2700 analyzer (immunoscattering turbidimetry), and an XE-5000 analyzer (electrical impedance optical method), respectively.

### 2.4. Statistical Analysis

SPSS 19.0 software (IBM, USA) was used to perform the statistical analysis. All data were tested for normality. Continuous data with a normal distribution was represented as the mean ± standard division (SD), and the *t*-test of independent samples was used for pairwise comparison, while continuous data that did not meet a normal distribution were subjected to the nonparametric rank-sum test. Skewed data was represented as median (quartiles) [*M*(*P*_25_–*P*_75_)]. After univariate analysis of the sepsis risk factors, factors with *P* < 0.05 were selected for multivariate logistic regression analysis (*P* < 0.05 was considered to be statistically significant). Multivariate analysis used multivariate nonconditional logistic regression and forward likelihood ratios were used with an entry probability of 0.05 and a rejection probability of 0.10. Following this, the odds ratio (OR) of the study factors, as well as the 95% confidence interval (CI), were calculated. The ROC curve analysis of data was performed and the area under the ROC curve (AUC) equaling 1.0 was considered to be the most reliable detection indicator. The diagnostic value was the lowest when the area under the ROC curve was between 0.5 and 0.7 and it was moderate when the area under the ROC curve was between 0.7 and 0.9. The diagnostic value was higher when the area under the ROC curve was above 0.9. The *Z* test was used to determine the area under the ROC curve. *P* < 0.01 was considered to be statistically significant.

## 3. Results

### 3.1. Basic Data of Newborns

The basic patient data for newborns is shown in Table 1 and includes gestational age, age in days after birth, birth weight, sex, type of delivery, and Apgar score. Among 192 infants, 100 were males (52.1%), and 92 were females (47.9%); the average gestational age is 38 (36.6–38) weeks; the cases of the premature delivery are 38 (19.8%) and the full-term ones are 154 (80.2%); the average age is 4 (1–28) days and the average birth weight 3050 g (2290–3450 g). In the control group, there were 106 children, of which 56 were males (accounting for 52.83%) and 50 were females (accounting for 47.16%), with an average age of 2 (1–28) days and average weight of 3150 g (2630–3450 g).

Compared with the control children, children with sepsis have shorter he gestational age and longer days after birth (*P* = 0.014 and *P* < 0.001). The birth weight and Apgar score were smaller in children with sepsis (*P* = 0.037 and *P* ≤ 0.001).

### 3.2. Bacterial Strains in Newborns with a Positive Blood Culture

There were 84 positive blood cultures in 192 newborns with sepsis. The predominant isolated strain was* G+ Streptococcus*, which accounted for 60% (50/84) of cases. The other isolated strain was by* G-bacilli*, which accounted for the remaining 40% (34/84) of sepsis cases (Table 2). A total of 34 strains were detected in premature infants, in which* G+ Streptococcus *was predominantly found in the bacterial culture. Among the* G+ Streptococcus*, 41% (14/34) were* Streptococcus* pneumoniae and 59% (20/34) were* G-bacilli* (Table 3).

### 3.3. Risk Factors for Neonatal Bacterial Sepsis

The results of univariate analysis displayed statistical significance in seven risk factors for neonatal bacterial sepsis including the age in days, birth weight, parity, gestational age, CRP, sCD14-ST, and TBIL value (*P* < 0.05). There was no correlation between the gender, type of birth, WBC, or percentage of granulocytes and neonatal bacterial sepsis (*P* > 0.05) (Table 4). According to the results of multivariate analysis, the age in days (OR = 1.176, *P* ≤ 0.001) can be considered as a relevant risk factor for sepsis (Table 5).

### 3.4. Prognostic Value of Risk Factors in Predicting Sepsis

**Figure 1:**
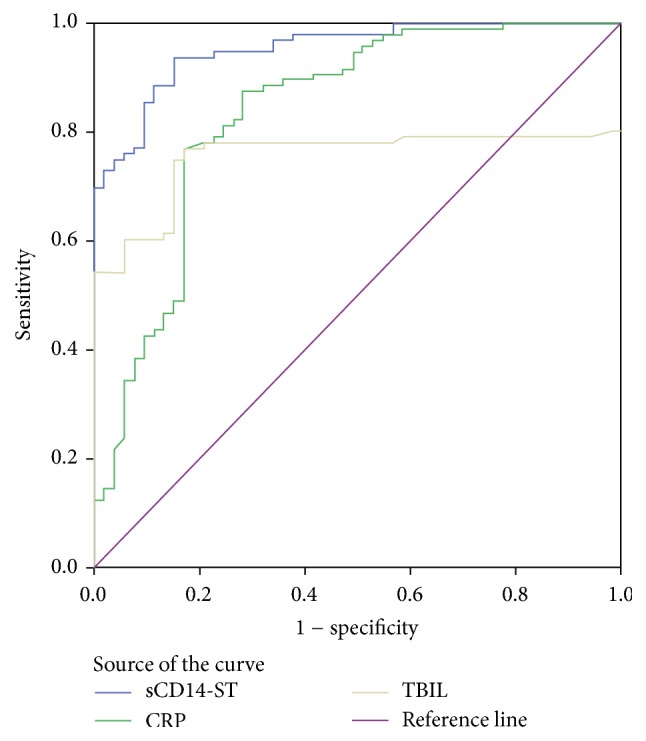
ROC curve of each detection indicator of the 192 pediatric patients with positive blood culture who suffered from sepsis.

Three statistically significant laboratory indicators (*P* < 0.05) in univariate analysis were used to plot the ROC curve, including the CRP, sCD14-ST, and TBIL value. The prognostic value of each risk factor for sepsis was analyzed. The results have shown that, compared with the area underneath the curve, ROC^AUC^ of sCD14-ST, CRP, and TBIL were all statistically significant (*P* < 0.01). The highest value of ROC^AUC^ was sCD14-ST (0.953), followed by CRP and TBIL. The comparisons among ROC^AUC^ of sCD14-ST, CRP, and TBIL were all statistically significant (*P* < 0.01, Figure 1 and Table 6).

## 4. Discussion

Clinical manifestations of neonatal sepsis were different in studied cases, so it is a difficult clinical pediatric problem. In addition to laboratory diagnostic indicators, the mother-child factors, as well as the hospital environment, are the typical causes of sepsis [10–12]. Our study found that the top three bacterial strains causing neonatal sepsis are* Escherichia coli, Staphylococcus aureus, *and* Streptococcus agalactiae*, which was consistent with international studies [13–17]. However, a study by Chen and Jiang found 73% of full-term infants to be infected with Streptococcus [18]. This might be associated with a difference in the distribution of regional microorganisms, the internal mother-child factors, or a limited number of specimens.

This study also found that the main bacteria in premature infants were* Streptococcus pneumoniae* and* Escherichia coli*, which was in line with the findings of Fan and Li who associated sepsis with fetal factors and hospital infection [6, 19]. Premature infants are more likely to develop sepsis from primary SIRS due to their low birth weight, Jaundice Blu-ray treatment, immature development of various tissues and organs, prolonged and excessive use of antibiotics in the ICU, and contact with infections from equipment. Therefore, it is extremely important to have a good team in place for hospital infection management [20].

Infants resilience to infectious diseases is poor, so they are vulnerable to external bacterial invasion and SIRS, which can lead to multiple organ failure and sepsis [18, 21]. To date, there have been few published reports about the risk factors of neonatal sepsis at home and abroad [22, 23]. This study found seven statistically significant factors by using univariate analysis: the age in days, birth weight, parity, gestational age, CRP, sCD14-ST, and TBIL value (*P* < 0.05). But what needs to be pointed out is that the birth weight showed in our study was relatively higher comparing to the other studies about neonatal sepsis. However, our study showed that low birth weight and premature delivery were both risk factors of sepsis. This result needs to be proved by further bulk samples. It is known that the delivery route is also probably a risk factor, as* Streptococcus* sp. is acquired through vaginal delivery. But our result demonstrated that, between the sepsis group and the control group, the percentage is 58.3% versus 69.8% (*P* = 0.166). The biased statistics may be due to the low samples of patients.

By excluding potentially confounding factors, logistic regression revealed that age in days can be considered as an independent risk factor of neonatal sepsis (OR = 1.176, *P* ≤ 0.001). American researchers found that the incidence of pediatric sepsis was highest in infants younger than one year old and that it decreased when the age increases. They concluded that age was one of the factors influencing the pathogenesis of sepsis [7]. It was consistent with the results of univariate analysis in this study.

Currently, as a potential biomarker of infection, sCD14-ST is rarely reported at home and abroad [24, 25]. This study showed that ROC^AUC^ of sCD14-ST, CRP, and TBIL on sepsis are significantly different (*P* < 0.05). Moreover, our results indicated that the largest ROC^AUC^ of sCD14-ST was 0.953 with high sensitivity (93.8%) and specificity (77.1%) and suggested that sCD14-ST had a certain advantage in the early diagnosis of pediatric sepsis and higher sensitivity than CRP or TBIL. The results are consistent with the study of Su et al. [26].

Early-onset sepsis is mainly caused by maternal infection induced by the premature rupture of membranes and nonsterile delivery of mothers. Most isolated pathogens come from the mother's birth canal. Early-onset sepsis occurs within 72 hours after birth; its onset is acute and progresses rapidly and usually involves multiple organ systems. Late-onset sepsis is mainly caused by improper care of the umbilicus, skin infection, or meningitis. Late-onset sepsis often occurs within 72 hours after birth, and the condition is relatively mild. Due to a limited number of studied specimens, classified statistics for septic pediatric patients were not performed and regional differences in bacterial strains were not examined. A study conducted on a larger sample set would be needed to reach more specified conclusions about the influence of the physical state of the mother and infant on sepsis so as to analyze bacterial strains responsible for sepsis.

In summary, bacterial culture findings in neonatal sepsis (including sepsis in premature infants) were associated with local bacteria originating from mothers, children, and hospital, so the importance of improving hospital conditions should be emphasized. Multivariate logistic regression demonstrated that the age of newborns is an independent risk factor for sepsis. Therefore, timely monitoring of the aforementioned factors should be improved in the clinical setting to prevent the occurrence of neonatal sepsis. Moreover, sCD14-ST proved to be advantageous in the early diagnosis of sepsis.

## Figures and Tables

**Table 1:** The constituent ratio of basic data of newborns.

	The sepsis group (*n* = 192)	The control group (*n* = 106)	*P *value
Male	100 (52.1%)	56 (52.83%)	0.930
Gestational age (weeks)	38 (36.6–38.0)	39 (37.6–39.35)	0.014
Premature delivery (*n*)	38 (19.8%)	—	
Full term (*n*)	154 (80.2%)	106 (100%)	
Days after birth (day)	4 (1–28)	2 (1–28)	<0.001
Weight of birth (g)	3050 (2290–3450)	3150 (2630–3450)	0.037
<1500 g	2 (1.0%)	—	
<2000 g	14 (7.3%)	—	
<2500 g	20 (10.4%)	10 (9.4%)	
≥2500 g	156 (81.2%)	96 (90.6%)	
Nature of delivery			
Natural labour	112 (58.3%)	74 (69.8%)	0.166
Caesarean section	80 (41.7%)	32 (30.2%)	
Agar score	10 (9-10)	10 (10-10)	<0.001
4–7 points	4 (2.1%)	—	
8–10 points	188 (97.9%)	106 (100%)	

*Note*. All data were presented with the form of quartile or number of cases (%).

**Table 2:** The distribution and constituent ratio of pathogens in the 84 newborns with bacterial positive blood test (*n* = 84).

Pathogens	Number of cases (*n*)	Constituent ratio (%)
*Gram-positive bacteria*	50	59.5
* Staphylococcus aureus*	16	19.0
* Streptococcus agalactiae*	12	14.2
* Staphylococcus haemolyticus*	6	7.1
* Staphylococcus epidermidis*	4	4.8
* Streptococcus bovis type II*	4	4.8
* Staphylococcus saprophyticus*	2	2.4
* Streptococcus pneumoniae*	2	2.4
Other Streptococcus species	2	2.4
* Enterococcus faecium*	2	2.4
*Gram-negative bacteria*	34	40.5
* Escherichia coli*	24	28.6
* Salmonella Dublin*	4	4.8
* Serratia marcescens*	6	7.1

**Table 3:** The distribution and constituent ratio of pathogens in the 34 strains of preterm children with bacterial sepsis (*n* = 34 strains).

Pathogens	Number of cases (*n*)	Constituent ratio (%)
*Gram-positive bacteria*	14	41.0
* Streptococcus pneumoniae*	12	35.3
* Staphylococcus epidermidis*	2	5.9
*Gram-negative bacteria*	20	58.9
* Escherichia coli*	10	29.4
* Serratia marcescens*	6	17.6
* Salmonella Dublin*	4	11.8

**Table 4:** Univariate analysis of neonatal sepsis.

Groups	The sepsis group (*n* = 192)	The control group (*n* = 106)	*P *value
Male (%)	100 (52.1%)	56 (52.83%)	0.930
Number of days of birth (d)	4 (1–28)	2 (1–28)	<0.001
Parity (*n*)	2 (1–5)	2 (1–6)	0.004
Mode of birth, natural labour (%)	112 (58.3%)	74 (69.8%)	0.166
Gestational age (w)	38 (36.6–38)	39 (37.6–39.35)	0.014
Weight of birth (g)	3050 (2290–3450)	3150 (2630–3450)	0.036
WBC (G/L)	12.78 ± 5.74	13.01 ± 6.02	0.456
NEU (%)	51.70 ± 15.16	50.63 ± 19.63	0.710
CRP (mg/L)	18.08 (10.22–31.36)	0.63 (0.50–3.45)	<0.001
TBIL (mol/L)	214.40 (139.95–298.15)	71.65 (23.78–175.10)	<0.001
sCD14-ST (ng/L)	657.50 (455.25–885.00)	208.00 (139.50–347.50)	<0.001

*Note*. All data were presented with the form of quartile or number of cases (%). WBC (G/L) refers to the number of white blood cells, NEU (%) represents the percentage of neutrophils, and TBIL (mol/L) is total bilirubin.

**Table 5:** Unconditional logistic regression analysis of neonatal sepsis.

Relevant factors	OR value	95% CI	*P*
Number of days of birth (d)	1.176	1.106–1.25	<0.001
Parity (*n*)	0.692	0.481–0.994	0.046
Gestational age (w)	0.951	0.739–1.223	0.695
Weight (g)	0.788	0.34–1.828	0.579

*Note*. OR means odds ratio, and 95% CI refers to 95% confidence interval.

**Table 6:** ROC curve analysis and diagnostic efficacy evaluation of the three detection indicators in the 192 pediatric patients with sepsis.

Items	AUC	Diagnostic bounds	The Younger Index	Sensitivity (%)	Specificity (%)	SE	*P*	95% confidence interval
sCD14-ST	0.953^a^	304.5	0.787	93.8^a^	84.9^a^	0.015	<0.001	0.923~0.983
CRP	0.837	9.915	0.601	77.1	83.0	0.037	<0.001	0.765~0.909
TBIL	0.755	21.15	0.601	77.1	83.0	0.042	<0.001	0.674~0.837

*Note*. a represents comparison with TBIL and CRP, and *P* values were all less than <0.01; SE is the standard error, and *P* refers to the comparison between the area under the curve (ROC curve of diagnostic test without any diagnostic value is the area under the curve) and 0.5.
